# Validation of Six Nomograms for Predicting Non-sentinel Lymph Node Metastases in a Dutch Breast Cancer Population

**DOI:** 10.1245/s10434-015-4858-8

**Published:** 2015-09-14

**Authors:** Siem A. Dingemans, Peter D. de Rooij, Roos M. van der Vuurst de Vries, Leo M. Budel, Caroline M. Contant, Anne E. M. van der Pool

**Affiliations:** Department of Surgery, Maasstad Hospital Rotterdam, Rotterdam, The Netherlands; Department of Pathology, Maasstad Hospital Rotterdam, Rotterdam, The Netherlands

## Abstract

**Background:**

The usefulness of axillary lymph node dissection (ALND) in patients with positive sentinel nodes (SN) is still an ongoing debate. Several nomograms have been developed for predicting non-sentinel lymph node metastases (NSLNM). We validated six nomograms using data from 10 years of breast cancer surgery in our hospital.

**Methods:**

We retrospectively analyzed all patients with a proven breast malignancy and a SN procedure between 2001 and 2011 in our hospital.

**Results:**

Data from 1084 patients were reviewed; 260 (24 %) had a positive SN. No patients with isolated tumor cells, 6 patients (8 %) with micrometastases, and 65 patients (41 %) with macrometastases had additional axillary NSLNM. In 2 patients (3 %) with micrometastases, the ALND influenced postoperative treatment. In the group of patients with macrometastases tumor size >2 cm, extranodal growth and having no negative SNs were predictors of NSLNM. The revised MD Anderson Cancer Center and Helsinki nomograms performed the best, with an area under the curve value of 0.78.

**Conclusions:**

ALND could probably be safely omitted in most patients with micrometastases but is still indicated in patients with macrometastases, especially in patients with tumor size >2 cm, extranodal growth, and no negative SNs. The revised MD Anderson Cancer Center and Helsinki nomograms were the most predictive in our patient group.

In the Netherlands, women have an approximate 15 % lifetime risk of developing a breast malignancy.^1^ In 2013, a total of 14,000 new patients were diagnosed with invasive breast cancer.^2^ With a mortality of 3.8 % and substantial morbidity, it is responsible for a great burden to society.^1^

In the management of patients with invasive breast cancer, axillary lymph node status is an important determinant of prognosis. For nodal staging, axillary lymph node dissection (ALND) has, over time, been replaced by sentinel lymph node (SLN) biopsy (SLNB).^3^ Between 62 and 75 % of all patients will have a negative SLNB, and no further axillary treatment is indicated because it offers no advantage in survival.^3^^–^7 However, in patients with a positive sentinel node (SN), ALND has been the standard treatment until recent times. In case of isolated tumor cells in the SN, it is disproportional to perform an ALND.^8^ In cases of micro- and macrometastases in the SN, it is an ongoing discussion. It seems that in most patients, ALND is merely helpful in staging, rather than a treatment itself. Morbidity such as paresthesia in the forearm and axilla, persistent lymphedema, and operated arm weakness due to ALND could be avoided in a selected group of patients.^3^ Recent literature has showed that in patients with early breast cancer and limited SN involvement, ALND is not useful in gaining survival.^3^,^9^^–^11 For example, the American College of Surgeons Oncology Group Z0011 trial showed that among patients with limited SN metastatic breast cancer treated with breast conservation and systemic therapy, the use of SLNB alone compared to ALND did not result in inferior survival.^3^

ALND can be omitted in most patients if physicians are able to predict the axillary lymph node status by other means. Several nomograms have been developed for predicting non-sentinel lymph node metastases (NSLNM). The most widely validated nomograms^12^ are from Memorial Sloan Kettering Cancer Center (MSKCC), MD Anderson Cancer Center (MDA), the Mayo Clinics (Mayo), Tenon Hospital (Tenon), Cambridge Cancer Research, Stanford Cancer Center, and Helsinki University Central Hospital.^4^,^7^,^13^^–^17 The nomogram from MDA has recently been updated (MDA2).^18^

In this study, we retrospectively analyzed all patients in our hospital with SLNB-positive breast cancer to determine clinicopathologic factors that might help predict the involvement of NSLNM. Furthermore, we tried to validate the nomograms mentioned above.

## Materials and Methods

Eligible patients were those who underwent breast surgery between January 2001 and December 2011 in our hospital. Only patients with a proven malignancy and those who underwent a SLNB were included. We retrospectively reviewed patient charts. Patients with neoadjuvant chemotherapy were excluded, as were patients with a (synchronous) tumor of another origin. The following characteristics were noted: type of tumor; tumor size; multifocality; Bloom–Richardson grade; estrogen receptor, progesterone receptor, and HER2/neu status; lymphovascular invasion; number of negative and positive SNs collected; isolated tumor cells (<0.2 mm); micrometastases (>0.2 to ≤2 mm) or macrometastases (>2 mm) in the SN; extranodal growth; whether or not a complete ALND was performed; and the number of additional NSLNM in the ALND.

All patients underwent SLNB after injection of technetium-99 m 1 cm caudal to the areola. After induction, blue dye was injected intradermally 1 cm lateral to the areola; this combination is an accurate method of locating SNs.^19^ SNs were intraoperatively routinely analyzed by frozen section and postoperatively by hematoxylin and eosin staining and immunohistochemistry. The 7th edition tumor, node, metastasis classification system from the International Union against Cancer was used to stage the tumors.^20^ When patients had >3 positive NSLNM, they automatically received axillary radiotherapy.

### Nomograms

As noted above, the MSKCC, MDA, Mayo, Tenon, Cambridge, Stanford, and Helsinki nomograms are the most validated.^17^,^21^ We excluded the Cambridge and Mayo nomograms in our study because these nomograms require size of SLN metastases as a continuous variable. Unfortunately, these data were not available for a large group of patients in our study. All patients with a positive SLNB were evaluated in the four remaining nomograms as well as in the MDA2 nomogram. An online calculator was used for the MSKCC and MDA2 nomogram; the Stanford, Tenon, first MDA, and Helsinki nomograms were calculated by hand.

### Statistical Analysis

Categorical data are presented as percentage frequencies. Associations between the presence of NSLNM in ALND and the characteristics of our study group were analyzed by the *χ*^2^ test. Significance levels are derived from two-tailed tests and were set at *p* < 0.05. Multivariate analysis was performed using a logistical regression model to identify those risk factors independently associated with NSLNM that had been statistically significant in the univariate analysis. The mean predicted probability of NSLNM from the five nomograms was compared to our study group. Discrimination of the nomograms was assessed by calculating the area under the curve (AUC) of the receiver operating characteristic curve. It is widely accepted that AUC values between 0.7 and 0.8 represent considerable discrimination.^22^ Statistical analysis was performed by SPSS 20 (IBM, Armonk, NY).

## Results

A total of 1084 patients were eligible. Demographics of the study group are shown in Table 1. Of all patients, 260 (24 %) had a positive SN. Table 2 shows the percentages of isolated tumor cells, micrometastases and macrometastases, and percentages of ALND and NSLNM. Twenty-three patients (9 %) with a positive SN did not receive an ALND because of old age, short life expectancy, or isolated tumor cells in the SLNB results. In two patients (3 %) with micrometastases and NSLNM in the ALND, stage migration occurred. They received adjuvant axillary radiotherapy.

**Table 1:** Patient demographics

Characteristic	Value (*n* = 1084)
Age (years), median (range)	60 (26–92)
Tumor size	
Median (range) (mm)	17 (1–150)
≤20 mm	717 (66 %)
>20 mm	346 (32 %)
Not available	21 (2 %)
T stage	
pT1	604 (56 %)
pT2	359 (33 %)
pT3	28 (2 %)
pT4	11 (1 %)
pTis	82 (8 %)
Histomorphology	
Ductal carcinoma	859 (79 %)
Lobular carcinoma	99 (9 %)
Mixed type	6 (1 %)
Other	120 (11 %)
Bloom–Richardson grade	
Well differentiated	114 (11 %)
Moderately differentiated	570 (53 %)
Poorly differentiated	295 (27 %)
Not available	105 (9 %)
Estrogen receptor	
Positive	806 (74 %)
Negative	221 (20 %)
Not available	57 (6 %)
Progesterone receptor	
Positive	534 (49 %)
Negative	354 (33 %)
Not available	196 (18 %)
HER2/neu receptor	
Positive	162 (15 %)
Negative	605 (56 %)
Not available	317 (29 %)
Multifocality	
Yes	113 (10 %)
No	971 (90 %)

**Table 2:** Size of sentinel node metastases

Characteristic	*n* (%)	ALND	NSLNM
Isolated tumor cells	17 (7 %)	2 (12 %)	0 (0 %)
Micrometastases	83 (34 %)	77 (93 %)	6 (8 %)
Macrometastases	160 (62 %)	158 (99 %)	65 (41 %)

*ALND* axillary lymph node dissection, *NSLNM* non-sentinel lymph node metastases

**Fig. 1:**
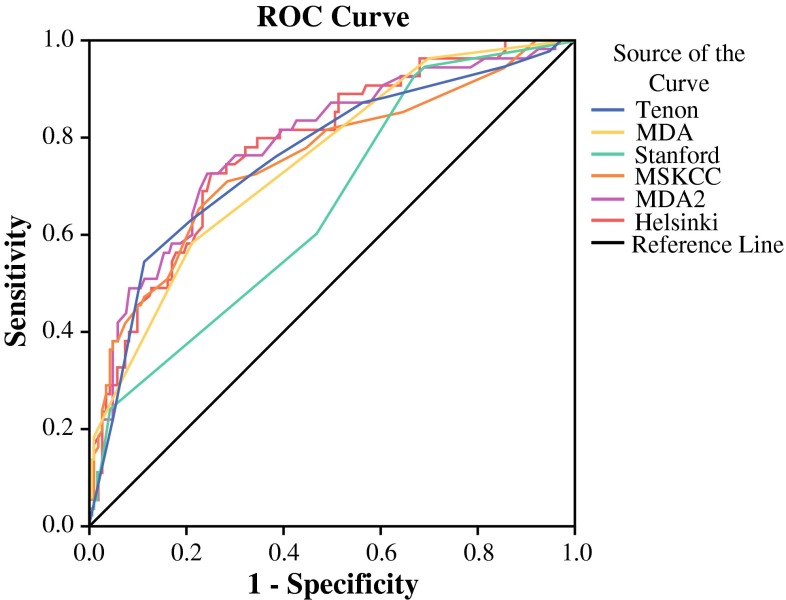
Receiver operating characteristic curve

In the micrometastases group, univariate analysis was performed; no significant predictors of NSLNM were found. In the univariate analysis of the patients with macrometastases (*n* = 158), statistically significant predictors of NSLNM were: age <50 years (*p* = 0.03), tumor size >2 cm (*p* = 0.003), lymphovascular invasion (*p* = 0.02), extranodal growth (*p* = 0.009), and having no negative SNs (*p* = 0.006, Table 3). In the multivariate analysis, tumor size of >2 cm, extranodal growth, and having no negative SNs remained statistically significant (Table 4). The AUC values for the MSKCC, MDA, Tenon, Stanford, Helsinki, and MDA2 nomograms were 0.72, 0.73, 0.76, 0.62, 0.78, and 0.78, respectively (Fig. 1).

**Table 3:** Univariate analysis of predictors of NSLNM in patients with macrometastases

Characteristic	NSLNM		*p* (2-tailed)
	No (*n* = 65)	Yes (*n* = 93)	
Age			0.03
≤50 years	25	28	
>50 years	68	37	
Multifocality			0.06
No	80	48	
Yes	13	17	
Tumor size			0.003
≤2 cm	38	12	
>2 cm	55	53	
Bloom–Richardson score			0.21
Well differentiated	13	4	
Moderately differentiated	44	32	
Poorly differentiated	31	28	
Estrogen receptor			0.45
Negative	17	15	
Positive	75	49	
Progesterone receptor			0.35
Negative	28	22	
Positive	52	29	
HER2/neu receptor			0.06
Negative	58	34	
Positive	13	17	
Lymphovascular invasion			0.02
No	86	52	
Yes	7	13	
Extranodal growth			0.009
No	76	41	
Yes	17	24	
No. of positive sentinel node collected			0.07
1	76	45	
>1	17	20	
No. of negative sentinel node collected			0.002
0	58	55	
≥1	35	10	

*NSLNM* non-sentinel lymph node metastases

**Table 4:** Multivariate analysis of predictors of non-sentinel lymph node metastases in patients with macrometastases

Factor	*p* (2-tailed)
Tumor size	
≤2 cm	1
>2 cm	2.6 (1.2–6.0) *p* = 0.02
Extranodal growth	
No	1
Yes	2.4 (1.1–5.4) *p* = 0.03
No. of negative sentinel nodes	
0	1
≥1	0.3 (0.1–0.7) *p* = 0.006

## Discussion

This study found that ALND will change postoperative treatment only in 3 % of patients with micrometastases, and that in patients with macrometastases, tumor size >2 cm, extranodal growth, and nonnegative SNs are predictors of NSLNM.

Recently the treatment of breast cancer has greatly changed. Whereas in the past standard treatment consisted of a radical mastectomy including an ALND, improved insights in cancer biology have resulted in less radical treatment.

By performing a SLNB, a large group of patients can be spared the adverse effects of ALND without lower survival rates.^18^ Currently, the standard treatment in our region for a patient with a positive SLNB, except for isolated tumor cells, is ALND. In 70 % of the patients with a positive SLNB in our study population, an ALND was performed in the absence of NSLNM, which is comparable to the 60–80 % described in literature.^18^ This is a serious matter, as these patients can experience significant adverse effects such as paresthesia, weakness of the treated arm, and lymphedema.^3^,^10^,^11^,^23^ Because of the comorbidities associated with ALND, it is always subject to discussion and its indication constantly revised and narrowed.

Our data support the current practice of not performing ALND in case of isolated tumor cells, as we did not find any NSLNM in patients with isolated tumor cells in the SN.

Performing ALND in all patients with micrometastases is currently under debate as well. There is an increasing amount of evidence suggesting that limited disease in non-sentinel lymph nodes has no effect on survival.^9^,^11^ The AATRM investigators showed in a randomized trial that in early breast cancer patients with micrometastases, SLNB alone was comparable to ALND in terms of locoregional control and distant disease. This practice had no significant effect on survival.^9^ The results of the IBCSG trial showed that there was no difference in survival between patients who did receive an ALND versus no axillary treatment when micrometastases were found in the SN. The authors of that trial advocated that ALND did not result in improved local control. Favorable long-term treatment events were significantly lower in the nonsurgical group. Additionally, there was no difference in the two groups receiving any type of adjuvant therapy.^11^ However, they only included patients with a clinically negative axilla, which should be taken into account when interpreting their results. Tvedskov et al., however, advocated that ALND should not be omitted in every patient with micrometastases, as they identified a subgroup of patients who have an increased risk of NSLNM and thus would benefit from ALND.^23^ Our results support both views; most of our patients (97 %) with micrometastases did not benefit from ALND, supporting the contemporary approach of omitting ALND in this patient group. However, 2 patients (3 %) did receive additional axillary radiotherapy; this was decided because of the number of NSLNM in the axilla.

One could opt to administer radiotherapy or routine follow-up with ultrasound to the axilla in case of micrometastases in the SLN instead of an ALND. In this way, all these patients can be spared the morbidity of an ALND. Chemotherapy and/or hormone therapy can be sufficient to eliminate remaining disease in patients with a low axillary tumor load, as suggested by the AATRM investigators.^9^

In patients with macrometastases >2 cm in size, extranodal growth and having no negative SNs proved to be predictors of NSLNM in our patient group. Although in most patients with macrometastases an ALND is indicated, more authors are now advocating a more conservative approach in this patient group as well; the Z0011 trial showed that in selected patients with T1 or T2 breast cancer and a positive SN, ALND might safely be omitted.^3^ All patients from the Z0011 trial, however, received whole breast irradiation, which is not a common practice in every center and thus should be taken in account. Additionally the Dutch AMAROS trial compared ALND to radiotherapy in T1–2 patients with a positive SLNB finding. They found similar results in terms of axillary control between the two treatments. The patients treated with ALND, however, experienced significantly more morbidities compared to patients treated with radiotherapy.^10^ We support the idea of narrowing the indication for ALND in patients with macrometastases as well. However, on the basis of our findings, we advocate a cautious strategy when dealing with a tumor >2 cm and/or extranodal growth and/or having no negative SNs.

 In our patient group, the MDA2 and Helsinki nomograms performed the best, with an AUC of 0.78. Compared to the earlier available nomogram from MDA, they added type of tumor, proportion of positive SN findings and extranodal growth. This resulted in an increase of 0.05 in the AUC (0.73–0.78). To our knowledge, this is only the second study to perform external validation of the revised MDA nomogram. This nomogram is available as an online calculator (http://www.mdanderson.org/), which is useful in common practice, and it might help determine treatment options in patients with a positive SLNB. Furthermore, the Helsinki nomogram performed equally well, with an AUC of 0.78. This nomogram is also user friendly: it is available in a free Excel form in which patient characteristics can be filled in. This nomogram might also be of use in decision making for SLNB-positive patients.

## Conclusions

In 6 patients (8 %) with micrometastases, NSLNM were found. In only 2 patients (3 %) did it have an impact on adjuvant treatment to the axilla. In patients with macrometastases, tumor size of >2 cm, extranodal growth, and having no negative SNs are predictors of NSLNM. The Helsinki and MDA2 nomograms proved to be the most predictive in our study group and are both easily usable.
